# Leisure-time physical activity participation and subjective class identity among Chinese residents: evidence from CGSS 2021

**DOI:** 10.3389/fsoc.2026.1846226

**Published:** 2026-09-21

**Authors:** Junming Ren, Chaochun Lang, Shaohong Pan

**Affiliations:** School of Physical Education and Health, Xichang University, Xichang, China

**Keywords:** CGSS, health sociology, leisure-time physical activity, social stratification, subjective class identity, subjective wellbeing

## Abstract

**Introduction:**

Subjective class identity is shaped not only by objective socioeconomic resources, but also by everyday experiences through which individuals evaluate their bodies, lives, and social relations. Although physical activity is often studied as a health behavior, leisure-time exercise, sports, and fitness may also function as socially meaningful lifestyle practices.

**Methods:**

Using data from the 2021 China General Social Survey, this study examines the association between regular leisure-time physical activity and subjective class identity among Chinese residents, and further assesses whether self-rated health, subjective well-being, and social interaction help account for this association. The baseline analytical sample includes 5,688 respondents, while pathway analyses use 3,802 respondents with complete information on self-rated health, subjective well-being, social interaction, and all covariates.

**Results:**

Ordered logit models show that regular physical activity is positively associated with higher subjective class identity after adjustment for demographic and socioeconomic covariates, including gender, age, marital status, household registration, ethnicity, region, education, political status, and personal income (*b* = 0.231, robust SE = 0.052, *p* < 0.001; OR = 1.26). The association remains stable across alternative measurements of subjective class identity and physical activity, ordered probit models, generalized ordered logit diagnostics, survey-weighted models, province fixed effects, clustered standard errors, propensity-score weighting, entropy balancing, and income-coding sensitivity analyses. Pathway models and Karlson-Holm-Breen decomposition indicate that self-rated health, subjective well-being, and social interaction jointly account for 73.20% of the total association in the pathway sample, with subjective well-being contributing the largest share. Heterogeneity analysis shows broadly positive associations across social groups, with the clearest subgroup difference appearing by marital status.

**Discussion:**

Given the cross-sectional design, the findings should be interpreted as robust associations and statistical pathways rather than causal effects. The study contributes to social stratification and health sociology by showing that subjective class identity is connected to embodied health, affective evaluation, and relational experiences embedded in everyday lifestyle practices.

## 1 Introduction

Social class is not only an objective location in a stratification system, but also a subjective judgment about where one stands in the social hierarchy. Objective class status is usually measured through income, education, occupation, housing, and other socioeconomic resources. Subjective class identity, by contrast, captures how people interpret their resources, life chances, social recognition, and prospects through everyday comparison. Classic and contemporary studies show that subjective social status does not necessarily correspond to objective status, and that this gap is shaped by material conditions, health, social relations, and evaluations of daily life (Jackman and Jackman, 1973; Han and Qiu, 2015; Xie, 2017).

Physical activity is often examined as a health behavior, but leisure-time exercise, sports, and fitness are also socially meaningful lifestyle practices. These activities are embedded in access to time, public space, community facilities, social networks, and cultural ideals of self-management. Physical inactivity also varies across national contexts and is associated with levels of human development (Dumith et al., 2011). From a stratification perspective, regular physical activity can signal self-discipline, health awareness, modernity, and the capacity to pursue a valued quality of life. In contemporary China, aspirations for a better life coexist with persistent inequalities in household registration, region, education, income, and access to public resources. Under these conditions, regular physical activity may be linked to how residents evaluate their social position, not only through health but also through wellbeing and social connectedness.

Most existing Chinese research has treated social class as an antecedent of sports and physical activity, examining how objective or subjective class position structures participation and related health outcomes (Li, 2017; Wang, 2018; Chen et al., 2023; Fang and An, 2024). A smaller body of research has begun to examine physical activity in relation to subjective class identity (Yang et al., 2022). Nevertheless, three issues remain insufficiently addressed: whether the association is observable in the general adult population after extensive adjustment for objective socioeconomic resources; whether it remains stable across alternative measurements, ordered-response specifications, survey weights, regional adjustments, and observed-selection checks; and the extent to which embodied health, affective wellbeing, and relational social interaction jointly account for this association within a common analytical framework.

Accordingly, this study uses CGSS 2021 data to examine whether regular leisure-time physical activity is associated with subjective class identity among Chinese residents and to assess three theoretically informed statistical pathways: self-rated health, subjective wellbeing, and social interaction. These three constructs are modeled as separate pathway variables rather than components of subjective class identity. The study makes three contributions. First, it shifts the analytical direction from the more established question of how class position structures physical activity to the less examined question of how physical activity is associated with subjective class self-placement. Second, it connects social stratification and health sociology by jointly evaluating embodied, affective, and relational pathways. Third, it tests the stability of the association across alternative measurements, ordered-response diagnostics, survey-weighted and region-adjusted models, observed-selection checks, and KHB decomposition, while maintaining a non-causal interpretation appropriate for cross-sectional data.

## 2 Theoretical framework and hypotheses

### 2.1 Physical activity as a stratified lifestyle practice

This study defines physical activity participation as free-time participation in exercise, fitness, or sports activities. This definition matters because leisure-time physical activity differs from physically demanding work or household labor. Leisure-time activity is more closely connected to lifestyle choice, body management, social visibility, and the unequal distribution of time and facilities. Subjective class identity refers to an individual's self-placement in the social hierarchy after considering objective living conditions, social comparison, perceived respect, and future expectations (Fang, 2008; Liang and Wang, 2023).

Several theoretical traditions help explain why physical activity may be associated with subjective class identity. Veblen's theory of leisure highlights the symbolic value of visible leisure practices (Veblen, 1953). Bourdieu's theory of distinction further suggests that tastes, bodily practices, and lifestyles are not merely individual preferences, but also resources through which social groups draw symbolic boundaries (Bourdieu, 1984). Shilling's discussion of physical capital similarly treats the body as a site where social advantage and disadvantage are accumulated and displayed (Shilling, 1991). Research on girls' soccer has examined embodied cultural capital in relation to social class, race and ethnicity, and sports performance (Goldsmith and Abel, 2024). These perspectives converge on one point: physical activity is not simply a biological behavior, but a social practice that may be related to how individuals evaluate themselves relative to others.

### 2.2 Physical activity and subjective class identity

Regular physical activity can be associated with subjective class identity in several ways. It may reflect the capacity to invest time and attention in the body, increase social visibility in public or community spaces, and support the perception that one is living a healthier and more modern life. Chinese studies have shown that physical activity participation is stratified by social resources and that sports participation is related to class-relevant outcomes (Li, 2017; Wang, 2018; Chen et al., 2023; Fang and An, 2024). However, fewer studies directly examine subjective class identity among Chinese residents. Based on the symbolic, embodied, and interactional meanings of regular physical activity, the first hypothesis is:

*H1: Regular leisure-time physical activity participation is positively associated with subjective class identity among Chinese residents*.

### 2.3 Embodied health pathway

The first mechanism is an embodied health pathway. Physical activity is consistently associated with better health outcomes, including higher self-rated health and better functional capacity (Piercy and Troiano, 2018; Bull et al., 2020; Wang et al., 2021). In stratification research, health is not only a biomedical condition but also an embodied resource. Better health can increase perceived control, reduce vulnerability, and strengthen the ability to participate in work, public life, and social comparison. These experiences may support a more favorable assessment of one's position in the social hierarchy (Wang, 2019; Fan, 2020; Feng et al., 2024). Therefore:

*H2: Health is a positive statistical pathway linking regular physical activity participation and subjective class identity*.

### 2.4 Affective wellbeing pathway

The second mechanism is an affective pathway through subjective wellbeing. Subjective wellbeing refers to an overall evaluation of life satisfaction and emotional experience (Diener, 1984). Physical activity can improve mood, reduce stress, and enhance life satisfaction (Chen et al., 2013; Liu, 2016; Zheng, 2019). Subjective wellbeing is relevant to class self-placement because people do not evaluate their social position only through income or occupation. They also consider whether their lives feel successful, manageable, and worthwhile. Higher subjective wellbeing may reduce feelings of relative deprivation and make respondents more likely to place themselves higher on a social ladder (Han and Qiu, 2015; Xu, 2023; Feng et al., 2024). Therefore:

*H3: Subjective wellbeing is a positive statistical pathway linking regular physical activity participation and subjective class identity*.

### 2.5 Relational social interaction pathway

The third mechanism is a relational pathway through social interaction. Physical activity often occurs in community parks, public squares, sports clubs, walking groups, gyms, and other shared settings. These settings create repeated opportunities for contact, reciprocity, and recognition. Social interaction matters for subjective class identity because class self-placement is relational: people evaluate themselves through reference groups, networks, and everyday recognition, not only through material resources. Prior research shows that physical activity may expand social participation and that social interaction is related to belonging and informal resources (He, 2016; Yang et al., 2021; Chen, 2024). Therefore:

*H4: Social interaction is a positive statistical pathway linking regular physical activity participation and subjective class identity*.

Taken together, these arguments yield an integrated framework in which regular leisure-time physical activity is associated with subjective class identity both directly and through three theoretically distinct but interrelated pathways: embodied health, affective wellbeing, and relational social interaction. Figure 1 summarizes this framework.

**Figure 1:**
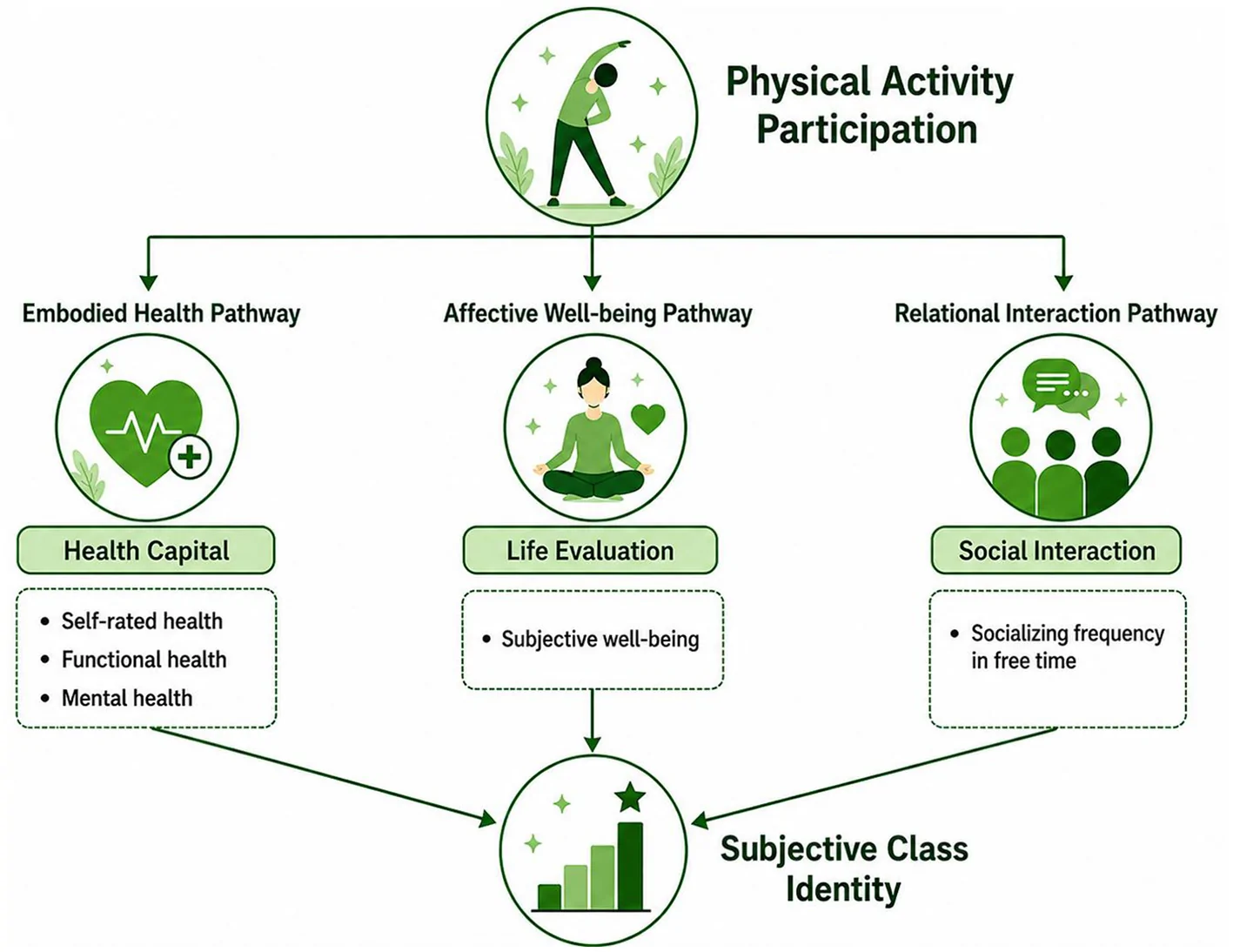
Conceptual framework of physical activity participation and subjective class identity. The solid arrows represent theoretically informed statistical pathways. Subjective class identity is measured independently using the 10-point social ladder; the pathway variables are analytically distinct from the outcome.

## 3 Data, variables, and analytical strategy

### 3.1 Data source and sample

The data come from the wave of the China General Social Survey (CGSS), a nationally representative survey administered by the China Survey and Data Center at Renmin University of China (China Survey and Data Center, 2023). The 2021 CGSS is particularly suitable for the present study because it measures leisure-time physical activity frequency, subjective class self-placement on a 10-point social ladder, self-rated health, subjective wellbeing, social interaction, and a broad set of demographic and socioeconomic characteristics within the same nationally representative survey. This combination permits the focal association and the three proposed statistical pathways to be evaluated within a common analytical framework. The selection of this cross-sectional wave therefore reflects its comprehensive construct coverage and measurement comparability. However, it does not permit temporal ordering, and all estimates are interpreted as associations and statistical pathways rather than causal effects. The raw CGSS 2021 file contains 8,148 respondents. Special missing-value codes, including refusal, do not know, and not applicable, were recoded as system missing for all study variables. Personal income values coded as special missing or negative were also set to missing. Eligibility for the baseline analysis required valid information on subjective class identity, regular physical activity participation, and all control variables. After list wise deletion, 2,460 respondents were excluded, leaving 5,688 respondents in the baseline analytical sample. The pathway analysis additionally required valid information on self-rated health, subjective wellbeing, and social interaction. A further 1,886 respondents were excluded, leaving 3,802 respondents in the pathway sample. The extended pathway analysis also required valid information on functional health and mental health. Nineteen additional respondents were excluded, leaving 3,783 respondents. Figure 2 summarizes the sample-selection process.

**Figure 2:**
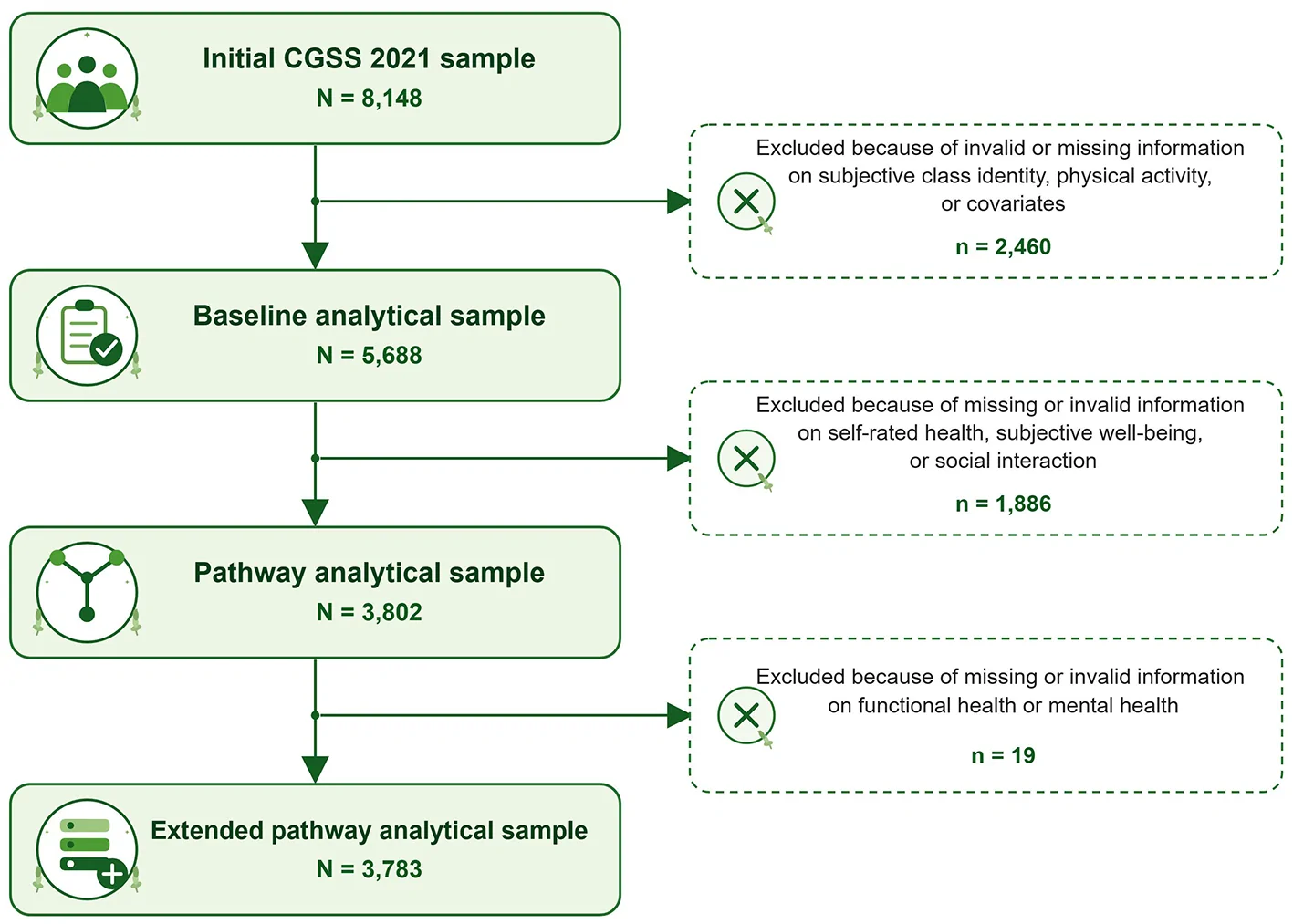
Flowchart of participant inclusion and analytical sample selection.

List wise deletion was used to keep the main models transparent and reproducible. As a separate sensitivity analysis, personal income was coded as ln (income + 1), which retained zero-income respondents and yielded an analytical sample of 7,078.

### 3.2 Measures

Subjective class identity is measured as a single-item outcome rather than a composite scale. It is assessed using CGSS item A43_a, on which respondents place themselves on a 10-point social ladder, with higher values indicating a higher perceived class position. The main ordered outcome combines adjacent categories into a five-category variable: 1–2 = lower, 3–4 = lower-middle, 5–6 = middle, 7–8 = upper-middle, and 9–10 = upper. This recoding reduces sparsity at the extremes and improves interpretability in ordered-response models. The original 10-point ladder is retained in robustness checks. Self-rated health, subjective wellbeing, and social interaction are modeled as separate pathway variables and are not components of the subjective class identity measure.

The key independent variable is regular leisure-time physical activity participation, measured by CGSS item A30_9. Respondents who reported participating in physical activity daily or several times a week are coded as regular participants. Respondents who reported participating several times a month, several times a year or less, or never are coded as non-regular participants. To address measurement concerns, the analysis also uses a three-category measure (never, occasional, regular) and a five-category frequency scale, where higher values indicate more frequent physical activity.

The pathway variables are self-rated health (A15), subjective wellbeing (A36), and social interaction in free time (A31_1). Higher values indicate better self-rated health, higher wellbeing, and more frequent social interaction. Extended pathway models additionally examine functional health (A16) and mental health (A17), both coded so that higher values indicate better health. Control variables include gender, age, age squared, marital status, household registration, ethnicity, region, education, political status, and log personal annual income. Table 1 summarizes the operationalization.

**Table 1:** Operationalization of variables.

Variable	CGSS item	Operationalization and coding
Subjective class identity	A43_a	Single-item outcome: 10-point social ladder recoded into five ordered categories: lower, lower-middle, middle, upper-middle, and upper. The original 10-point ladder is used in robustness checks.
Regular physical activity	A30_9	Daily or several times a week = 1; several times a month, several times a year or less, or never = 0.
Alternative physical activity measures	A30_9	Three-category measure: never, occasional, regular. Five-category frequency scale: higher values indicate more frequent physical activity.
Self-rated health	A15	Five-point scale, higher values indicate better self-rated health.
Functional health	A16	Five-point scale, higher values indicate better functional health; used in extended pathway models.
Mental health	A17	Five-point scale, higher values indicate better mental health; used in extended pathway models.
Subjective wellbeing	A36	Five-point scale, higher values indicate greater overall happiness.
Social interaction	A31_1	Frequency of socializing in free time; higher values indicate more frequent social interaction.
Gender	A2	Female = 1; male = 0.
Age	A3_1	Age in years, calculated as 2021 minus year of birth; age squared is included.
Marital status	A69	Married = 1; not married = 0.
Household registration	A18	Urban, non-urban, and other household registration.
Ethnicity	A4	Categorized as Han and non-Han.
Region	Province	Province names grouped into eastern, central, and western China.
Education	A7a	No education, primary/secondary, tertiary, postgraduate or above.
Political status	A10	CCP member = 1; non-CCP = 0.
Personal income	A8a	Log of positive personal annual income. Sensitivity analysis uses ln(income + 1).

SCI, subjective class identity; PA, physical activity. Subjective class identity is a single-item outcome. Self-rated health, subjective wellbeing, and social interaction are separate pathway variables and are not used to construct the outcome. Higher values for health, subjective wellbeing, and social interaction indicate more favorable levels.

### 3.3 Analytical strategy

Because the main dependent variable is ordinal, the primary specification is an ordered logit model with robust standard errors. The model estimates the association between regular physical activity participation and the cumulative odds of reporting a higher level of subjective class identity. The main specification adjusts for gender, age, age squared, marital status, household registration, ethnicity, region, education, political status, and log personal income. All analyses were conducted in Stata 19.

The empirical strategy proceeds in four steps. First, baseline ordered logit models estimate the association between physical activity participation and subjective class identity. Second, measurement robustness is examined by using an ordered probit model, the original 10-point class ladder, a three-category physical-activity measure, and a five-category physical-activity frequency measure. Third, the proportional-odds assumption is assessed using generalized ordered logit models with the autofit procedure (McCullagh, 1980; Williams, 2006). Fourth, pathway models estimate the association between physical activity and each pathway variable and then add self-rated health, subjective wellbeing, and social interaction to the ordered logit outcome model. To address the scale-comparability problem in nested non-linear probability models, the study further uses the Karlson-Holm-Breen (KHB) decomposition method. The KHB method separates the change in the physical-activity coefficient attributable to observed pathway variables from rescaling bias and expresses the total, direct, and explained components on a common ordered-logit scale (Kohler et al., 2011; Karlson et al., 2012). Because the data are cross-sectional, the decomposition is interpreted as statistical pathway evidence rather than causal mediation.

Additional robustness checks address concerns about sample weights, model choice, and observed selection. The main models are unweighted because the goal is to estimate conditional associations net of major design-related covariates. Survey-weighted ordered logit models using the CGSS raking weight and the basic weight are reported as robustness checks (Solon et al., 2015). Models with province fixed effects, province-clustered standard errors, propensity-score weighting, and entropy balancing are also estimated (Rosenbaum and Rubin, 1983; Hainmueller, 2012).

An instrumental-variable strategy was carefully considered. The CGSS includes a neighborhood-environment item indicating whether the residential area is suitable for physical activity. Although this item predicts physical activity participation, it is also directly associated with subjective class identity after covariate adjustment. It is therefore unlikely to satisfy the exclusion restriction because perceived neighborhood quality, public-service provision, and local status comparison may directly shape class self-placement. In the absence of a defensible external instrument, this study does not report IV estimates and interprets all findings as associations.

## 4 Results

### 4.1 Descriptive statistics

Table 2 presents descriptive statistics for the baseline analytical sample. The mean of the five-category subjective class identity variable is 2.48, and the mean of the original 10-point ladder is 4.34, suggesting that respondents on average locate themselves around the lower-middle to middle range of the subjective social hierarchy. This distribution is consistent with previous studies showing that subjective class self-placement in China tends to be concentrated in the middle and lower-middle categories rather than at the two extremes (Xu, 2023; Hu and Ren, 2021).

**Table 2:** Descriptive statistics for the baseline analytical sample.

Variable	*N*	Mean or %	SD	Range/category detail
Subjective class identity, 5-category	5,688	2.48	0.93	1–5
Subjective class identity, 10-point	5,688	4.34	1.86	1–10
Regular physical activity	5,688	41.75%	NA	Non-regular 58.25%, regular 41.75%
Physical activity, 3-category	5,688	NA	NA	Never 32.56%, occasional 25.69%, regular 41.75%
Physical activity, 5-category	5,688	2.91	1.61	1–5
Self-rated health	5,687	3.50	1.06	1–5
Functional health	5,674	3.97	1.20	1–5
Mental health	5,673	3.99	1.05	1–5
Social interaction	5,685	2.63	1.10	1–5
Subjective wellbeing	3,806	4.00	0.79	1–5
Female	5,688	49.31%	NA	Male 50.69%, female 49.31%
Age	5,688	52.56	16.57	18–95
Married	5,688	73.00%	NA	Not married 27.00%, married 73.00%
Household registration	5,688	NA	NA	Urban 34.46%, non-urban 64.33%, other 1.21%
Han ethnicity	5,688	93.58%	NA	Non-Han 6.42%, Han 93.58%
Region	5,688	NA	NA	Eastern 43.97%, central 29.36%, western 26.67%
Education	5,688	NA	NA	No education 9.09%, primary/secondary 69.30%, tertiary 20.15%, postgraduate+ 1.46%
CCP member	5,688	14.61%	NA	Non-CCP 85.39%, CCP member 14.61%
Log personal income	5,688	10.13	1.37	2.996–16.117

Descriptive statistics are based on the baseline analytical sample unless a variable has additional item nonresponse. Percentages are un weighted. The pathway sample is smaller mainly because subjective wellbeing has additional missing values. SCI = subjective class identity; PA = physical activity.

Regarding physical activity participation, 41.75% of respondents report engaging in regular leisure-time physical activity, while 32.56% report never participating. This indicates that leisure-time physical activity is not a marginal behavior in the sample, but there remains substantial variation in participation frequency. The three-category and five-category physical activity measures further show that the analysis is not solely dependent on a binary distinction between participants and non-participants, which helps reduce concerns about information loss in the main independent variable.

The sample is relatively balanced by gender, with 49.31% female respondents. The mean age is 52.56 years, and 73.00% of respondents are married. In terms of household registration, 64.33% of respondents have non-urban household registration, while 34.46% have urban household registration. Regionally, 43.97% of respondents are from eastern China, 29.36% from central China, and 26.67% from western China. Educational attainment is concentrated in the primary/secondary category, while respondents with postgraduate education account for only 1.46% of the sample. These characteristics suggest that the sample covers diverse social groups, but also reflects the demographic structure of the CGSS adult population.

For the proposed pathway variables, the mean self-rated health score is 3.50, while functional health and mental health have slightly higher means of 3.97 and 3.99, respectively. The mean subjective wellbeing score is 4.00, indicating a generally favorable level of self-reported happiness among respondents with valid responses. Social interaction has a mean of 2.63, suggesting moderate variation in free-time socializing frequency. However, subjective wellbeing is available for fewer respondents than the other pathway variables, which explains the smaller pathway sample used in the subsequent pathway analysis. Therefore, the pathway results should be interpreted with attention to this reduction in sample size.

### 4.2 Baseline and measurement robustness

Table 3 reports the baseline models and measurement-robustness checks. In the model without controls, regular leisure-time physical activity participation is positively associated with subjective class identity (*b* = 0.379, robust SE = 0.050, *p* < 0.001). After adjusting for gender, age, age squared, marital status, household registration, ethnicity, region, education, political status, and log personal income, the coefficient decreases but remains positive and statistically significant (*b* = 0.231, robust SE = 0.052, *p* < 0.001). This attenuation suggests that part of the bivariate association is related to demographic and socioeconomic differences between regular and non-regular participants. Nevertheless, the persistence of a significant coefficient after full adjustment indicates that regular physical activity remains positively associated with subjective class identity net of these observed characteristics.

**Table 3:** Baseline and measurement-robustness models.

Specification	Outcome and PA measure	Key estimate	*N*	Interpretation
Model 1: ordered logit, no controls	SCI5/regular PA	*b* = 0.379 (0.050)^***^	5,688	Positive bivariate association
Model 2: ordered logit, full controls	SCI5/regular PA	*b* = 0.231 (0.052)^***^; OR = 1.26	5,688	Main adjusted association
Ordered probit	SCI5/regular PA	*b* = 0.128 (0.030)^***^	5,688	Model-form robustness
Original class ladder	SCI10/regular PA	*b* = 0.218 (0.050)^***^	5,688	Dependent-variable robustness
Three-category PA	SCI5/occasional vs never	*b* = 0.214 (0.068)^**^	5,688	Occasional participation is positive
Three-category PA	SCI5/regular vs never	*b* = 0.334 (0.064)^***^	5,688	Regular participation is larger
Five-category PA frequency	SCI5/frequency scale	*b* = 0.087 (0.017)^***^	5,688	Higher frequency is positive

SCI5, five-category subjective class identity; SCI10, original 10-point subjective class identity; PA, physical activity. Robust standard errors are in parentheses. All controlled models include gender, age, age squared, marital status, household registration, ethnicity, region, education, political status, and log personal income. ^**^p < 0.01, ^***^p < 0.001.

In odds-ratio terms, regular participants have 1.26 times the odds of reporting a higher subjective class identity than non-regular participants, holding the control variables constant. This result supports H1, but it should be interpreted as an adjusted association rather than a causal effect because the data are cross-sectional.

The association is not driven by the choice of model specification or by the recoding of key variables. The coefficient remains positive and statistically significant in an ordered probit model, indicating that the finding is not specific to the ordered logit functional form. It also remains robust when the original 10-point subjective class ladder is used instead of the five-category recoded outcome, which helps address concerns about information loss in the dependent variable.

The results are also stable across alternative measurements of physical activity. In the three-category specification, both occasional and regular participants report higher subjective class identity than respondents who never participate, and the coefficient for regular participation is larger than that for occasional participation. The five-category frequency measure further shows a positive frequency gradient, suggesting that more frequent physical activity participation is associated with higher subjective class self-placement. Taken together, these measurement checks indicate that the main finding is not an artifact of dichotomizing physical activity participation or recoding subjective class identity.

### 4.3 Model diagnostics and robustness checks

Table 4 summarizes the model diagnostics and additional robustness checks. The generalized ordered logit diagnostic indicates that the proportional-odds constraint is tenable for the key physical-activity variable. The constraint test for regular physical activity is statistically insignificant in the auto fit procedure (*p* = 0.747), and the final partially constrained model does not reject the parallel-lines assumption overall (chi-square = 28.47, df = 27, *p* = 0.387). These results support the appropriateness of the ordered logit specification for the main analysis.

**Table 4:** Model diagnostics and additional robustness checks.

Diagnostic or robustness check	Key result	Sample	Conclusion
Generalized ordered logit (autofit)	Parallel-lines test: chi-square(27) = 28.47, p = 0.387; PA constraint: p = 0.747	5,688	Core PA variable satisfies the proportional-odds constraint; the final partially constrained model does not reject parallel lines.
Province fixed effects	b = 0.239 (0.052)^***^	5,688	Positive association remains.
Province-clustered standard errors	b = 0.231 (0.058)^***^	5,688	Inference remains significant.
Raking-weighted ordered logit	b = 0.314 (0.088)^***^	5,688	Positive association remains under survey weighting.
Basic-weighted ordered logit	b = 0.265 (0.058)^***^	5,688	Positive association remains.
Propensity-score weighted ordered logit	b = 0.295 (0.074)^***^	3,863	Observed-selection check is consistent.
Entropy-balanced ordered logit	b = 0.241 (0.053)^***^	5,688	Covariate-balance check is consistent.
Income coding with ln (income + 1)	b = 0.270 (0.046)^***^	7,078	Result is robust when zero-income cases are retained.

Each coefficient refers to regular physical activity participation unless otherwise stated. Robust standard errors are in parentheses. Weighted models use available positive survey weights. PA = physical activity. ^***^p < 0.001.

The positive association between regular physical activity and subjective class identity remains stable across several alternative specifications. When province fixed effects are included, the coefficient remains positive and statistically significant (*b* = 0.239, robust SE = 0.052, *p* < 0.001). The result is also unchanged when standard errors are clustered at the province level (*b* = 0.231, SE = 0.058, *p* < 0.001), suggesting that the statistical inference is not sensitive to provincial clustering.

Survey-weighted models produce similar results. The coefficient remains positive and statistically significant in both the raking-weighted ordered logit model (*b* = 0.314, robust SE = 0.088, *p* < 0.001) and the basic-weighted ordered logit model (*b* = 0.265, robust SE = 0.058, *p* < 0.001). These estimates suggest that the main association is not dependent on the use of un weighted models.

The association also remains positive in models that adjust for observable differences between regular and non-regular participants. The propensity-score weighted model yields a coefficient of 0.295 (robust SE = 0.074, *p* < 0.001), while the entropy-balanced model yields a coefficient of 0.241 (robust SE = 0.053, *p* < 0.001). These results are consistent with the baseline finding after reweighting the sample on observed covariates.

Finally, the income-coding sensitivity analysis shows that the result is not dependent on excluding zero-income respondents. When personal income is coded as ln (income + 1), thereby retaining zero-income cases and increasing the sample size to 7,078, the coefficient for regular physical activity remains positive and statistically significant (*b* = 0.270, robust SE = 0.046, *p* < 0.001). Overall, the diagnostic tests and robustness checks indicate that the main association is stable across model form, weighting strategy, regional adjustment, observed-selection adjustment, and income-coding choices.

### 4.4 Pathway analysis

Table 5 reports the pathway models. Regular physical activity is positively associated with all three proposed pathway variables. Specifically, regular participants report better self-rated health (*b* = 0.167, robust SE = 0.033, *p* < 0.001), higher subjective wellbeing (*b* = 0.162, robust SE = 0.026, *p* < 0.001), and more frequent social interaction (*b* = 0.205, robust SE = 0.037, *p* < 0.001) than non-regular participants. These patterns are consistent with prior research linking physical activity to health benefits, subjective wellbeing, and social connectedness (Piercy and Troiano, 2018; Bull et al., 2020; Buecker et al., 2021; Eime et al., 2013).

**Table 5:** Pathway models.

Model	Dependent variable	Key estimate	N	Interpretation
A-path 1	Self-rated health	Regular PA: *b* = 0.167 (0.033)^***^	3,802	Regular PA is associated with better health.
A-path 2	Subjective wellbeing	Regular PA: *b* = 0.162 (0.026)^***^	3,802	Regular PA is associated with higher wellbeing.
A-path 3	Social interaction	Regular PA: *b* = 0.205 (0.037)^***^	3,802	Regular PA is associated with more frequent interaction.
Outcome with pathways	SCI5	Regular PA: *b* = 0.049 (0.065), *p* = 0.450	3,802	Direct association is strongly attenuated
Outcome with pathways	SCI5	Self-rated health: *b* = 0.210 (0.035)^***^	3,802	Health is positively associated with SCI.
Outcome with pathways	SCI5	Subjective wellbeing: *b* = 0.514 (0.046)^***^	3,802	Wellbeing is the largest pathway coefficient.
Outcome with pathways	SCI5	Social interaction: *b* = 0.075 (0.030)^*^	3,802	Interaction is positively associated with SCI.

The first three models are linear regressions for pathway variables. The outcome model is an ordered logit model predicting SCI5. All models include the full control set. SCI = subjective class identity. Robust standard errors are in parentheses. ^*^p < 0.05, ^***^p < 0.001.

When self-rated health, subjective wellbeing, and social interaction are included simultaneously in the ordered logit outcome model, all three pathway variables are positively associated with subjective class identity. Subjective wellbeing has the largest coefficient among the three pathway variables (*b* = 0.514, robust SE = 0.046, *p* < 0.001), followed by self-rated health (b = 0.210, robust SE = 0.035, *p* < 0.001) and social interaction (*b* = 0.075, robust SE = 0.030, *p* < 0.05). After these pathway variables are included, the coefficient for regular physical activity declines substantially and becomes statistically insignificant (*b* = 0.049, robust SE = 0.065, *p* = 0.450). This pattern suggests that the association between regular physical activity and subjective class identity is largely accounted for by differences in health, wellbeing, and social interaction in the pathway sample.

Table 6 further reports the KHB decomposition for the pathway sample. In the reduced model, which excludes the three pathway variables but retains the full set of controls, the coefficient for regular physical activity is positive and statistically significant (*b* = 0.183, robust SE = 0.064, *p* = 0.004). In the full model, after self-rated health, subjective wellbeing, and social interaction are added, the direct association becomes smaller and statistically insignificant (*b* = 0.049, robust SE = 0.065, *p* = 0.450). The total explained component is 0.134 (robust SE = 0.020, *p* < 0.001), accounting for 73.20% of the total association.

**Table 6:** KHB decomposition of pathway associations in ordered logit models.

Component	Coefficient (robust SE)	*p*-value	Share of total association (%)	Share of explained component (%)
Total association/reduced model	0.183 (0.064)	0.004	100.00	–
Direct association/full model	0.049 (0.065)	0.450	26.80	–
Total explained component	0.134 (0.020)	< 0.001	73.20	100.00
Self-rated health component	0.035 (0.009)	–	19.18	26.21
Subjective wellbeing component	0.083 (0.015)	–	45.63	62.33
Social interaction component	0.015 (0.007)	–	8.39	11.46

KHB, Karlson-Holm-Breen; PA, physical activity; SCI, subjective class identity. Estimates are based on ordered logit models using the pathway sample (*N* = 3,802). The full model includes self-rated health, subjective wellbeing, social interaction, and all control variables. The reduced model excludes the three pathway variables but keeps the same covariates. Shares of individual pathway components refer to their percentage contribution to the explained component and to the reduced-model association. Estimates are interpreted as statistical pathway decompositions rather than causal mediation effects because the data are cross-sectional.

Among the three pathway variables, subjective wellbeing contributes the largest share of the explained component. It accounts for 62.33% of the explained component and 45.63% of the total association. Self-rated health accounts for 26.21% of the explained component and 19.18% of the total association, while social interaction accounts for 11.46% of the explained component and 8.39% of the total association. These results suggest that the affective pathway plays the most prominent role, while the embodied health and relational interaction pathways also contribute to the overall association.

### 4.5 Heterogeneity

Table 7 presents subgroup estimates and the corresponding interaction results. Overall, the association between regular physical activity and subjective class identity is positive in most subgroups. The coefficient is statistically significant among both men and women, among urban and non-urban respondents, among unmarried and married respondents, among respondents in eastern and western China, and among those with primary/secondary or tertiary education. These results suggest that the positive association is not confined to a single segment of the sample.

**Table 7:** Heterogeneity analysis.

Dimension	Subgroup	PA coefficient (SE)	*N*	Interaction evidence
Gender	Male	0.256 (0.073)^***^	2,883	Female x PA: −0.057, *p* = 0.572
Gender	Female	0.197 (0.074)^**^	2,805	No significant gender difference
Household registration	Urban	0.363 (0.087)^***^	1,960	Non-urban x PA: −0.142, *p* = 0.176
Household registration	Non-urban	0.169 (0.066)^*^	3,659	No significant household-registration difference
Marital status	Not married	0.386 (0.099)^***^	1,536	Married x PA: −0.231, *p* = 0.041
Marital status	Married	0.167 (0.062)^**^	4,152	Association is stronger among unmarried respondents
Region	Eastern	0.226 (0.077)^**^	2,501	Central x PA: −0.069, *p* = 0.563; Western x PA: 0.055, *p* = 0.667
Region	Central	0.178 (0.099)+	1,670	No significant regional difference
Region	Western	0.298 (0.104)^**^	1,517	No significant regional difference
Education	No education	0.250 (0.187)	517	Education interactions are not significant
Education	Primary/secondary	0.204 (0.063)^**^	3,942	Positive association
Education	Tertiary	0.351 (0.118)^**^	1,146	Positive association
Education	Postgraduate+	−0.243 (0.504)	83	Small subgroup; unstable estimate

Each subgroup model uses ordered logit with robust standard errors and the relevant control variables. + *p* < 0.10, ^*^p < 0.05, ^**^p < 0.01, ^***^p < 0.001.

At the same time, the formal interaction tests show that most subgroup differences are limited. The interaction terms for gender, household registration, region, and education are not statistically significant, indicating that the magnitude of the association does not differ systematically across these dimensions. Although the subgroup coefficients vary in size, these differences should be interpreted cautiously in the absence of statistically significant interaction effects.

The clearest heterogeneity appears by marital status. The interaction between regular physical activity and being married is negative and statistically significant (*b* = −0.231, *p* = 0.041), indicating that the positive association between regular physical activity and subjective class identity is stronger among respondents who are not married than among those who are married. Subgroup estimates are consistent with this pattern: the coefficient for regular physical activity is 0.386 (SE = 0.099, *p* < 0.001) among unmarried respondents, compared with 0.167 (SE = 0.062, *p* < 0.01) among married respondents. This difference may be related to the fact that marital status structures time use, social roles, household responsibilities, and lifestyle routines. Previous studies have shown that marital status and partner-related factors are associated with physical activity participation, and that spousal or partner support can shape activity behavior (Sobal and Hanson, 2010; Pettee et al., 2006; Yuan et al., 2024).

The education results also warrant caution. Although the coefficients are positive for respondents with primary/secondary and tertiary education, the interaction terms are not statistically significant, and the postgraduate subgroup is very small (*N* = 83). The estimate for this subgroup is therefore unstable and should not be over interpreted.

Figures 3, 4 further visualize the marital-status interaction by presenting adjusted predicted probabilities for the upper-middle and upper subjective class categories. The increase associated with regular physical activity is steeper among respondents who are not married, which is consistent with the negative interaction coefficient for married respondents reported in Table 7.

**Figure 3:**
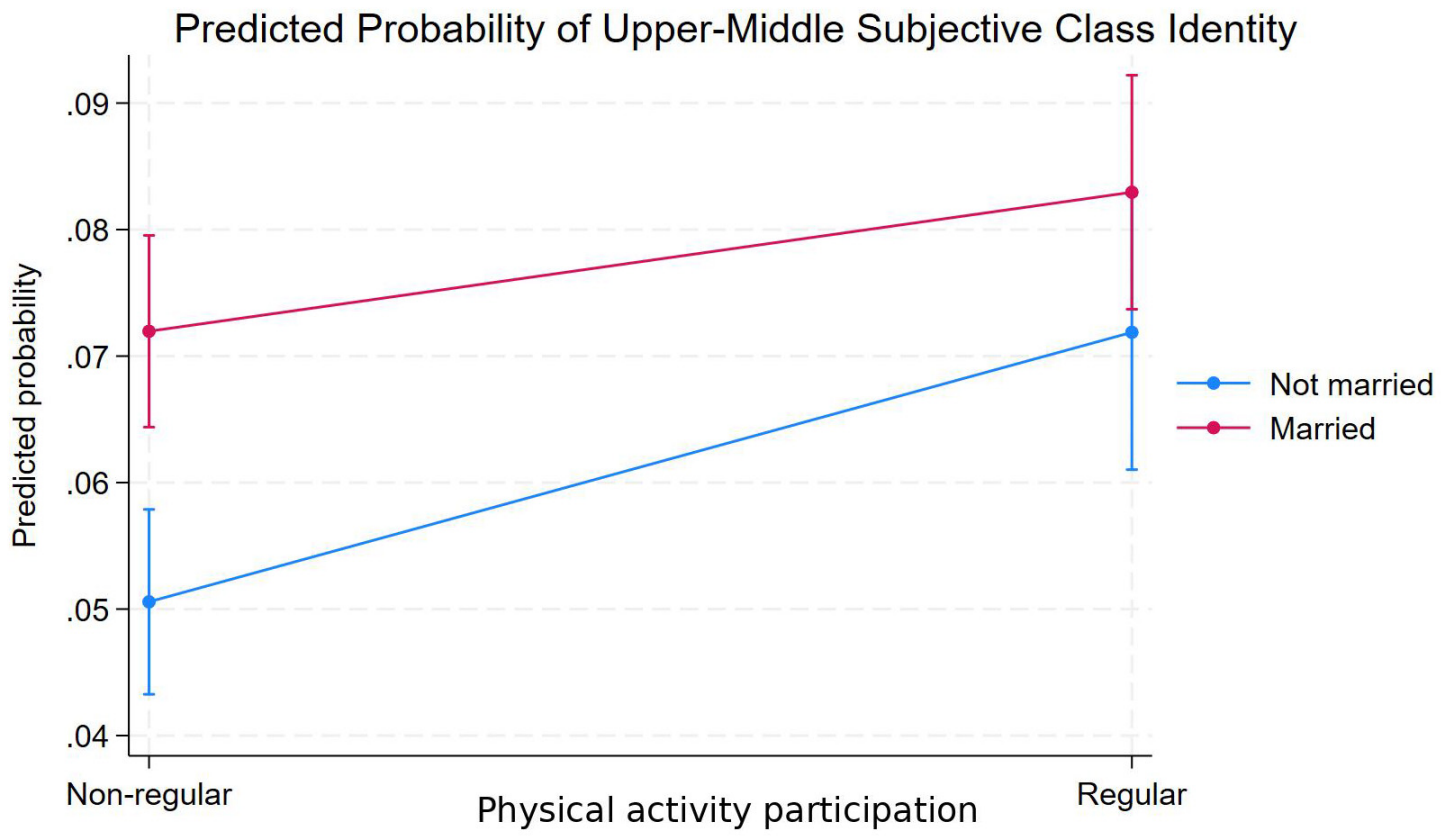
Adjusted predicted probability of upper-middle subjective class identity by physical activity participation and marital status.

**Figure 4:**
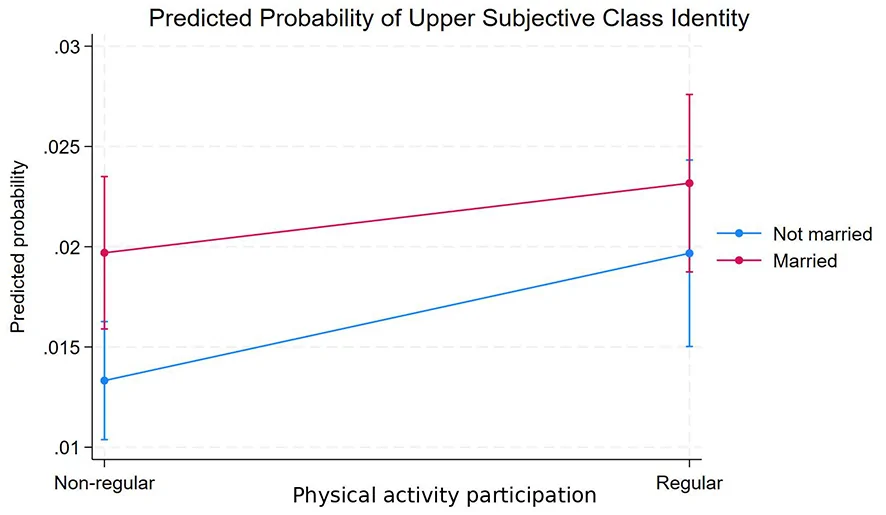
Adjusted predicted probability of upper subjective class identity by physical activity participation and marital status.

## 5 Discussion

### 5.1 Contributions to stratification and health sociology

This study contributes to social stratification research by showing that subjective class identity is related not only to conventional socioeconomic indicators such as income, education, household registration, and political status, but also to everyday lifestyle practices. Previous studies have shown that subjective social status does not always correspond directly to objective class position and that class self-placement is shaped by social comparison, perceived resources, and everyday life experiences (Jackman and Jackman, 1973; Han and Qiu, 2015; Xie, 2017). Regular leisure-time physical activity remains positively associated with higher subjective class self-placement after extensive covariate adjustment and across multiple robustness checks. This finding suggests that subjective class identity is not formed solely through objective material resources, but also through how individuals experience their bodies, evaluate their lives, and participate in social relations.

The present findings extend Chinese research on the stratification of sports participation and on the relationship between physical activity and subjective class identity (Li, 2017; Wang, 2018; Yang et al., 2022; Fang and An, 2024). Their added value lies in testing this relationship in a national adult sample under extensive socioeconomic adjustment, multiple measurement and model specifications, and a common pathway-decomposition framework. The finding does not imply that exercise can replace structural resources or overcome social inequality. Rather, it suggests that lifestyle practices may form part of the everyday process through which individuals interpret their position in the social hierarchy.

The study also contributes to health sociology by treating physical activity as a social practice rather than only a health behavior. The observed association is consistent with theories of distinction, physical capital, and lifestyle-based stratification (Bourdieu, 1984; Shilling, 1991; Stempel, 2005; Warde, 2006). Leisure-time physical activity may carry symbolic meanings related to health consciousness, self-discipline, social participation, and the pursuit of a better quality of life. In the Chinese context, where social mobility, public service access, and lifestyle differentiation are closely intertwined, these meanings may be relevant to how residents evaluate their class position even after objective socioeconomic characteristics are considered.

### 5.2 Interpreting the pathway findings

The pathway analysis provides further insight into how regular physical activity is linked to subjective class identity. After self-rated health, subjective wellbeing, and social interaction are added to the model, the coefficient for regular physical activity declines substantially and becomes statistically insignificant. The KHB decomposition shows that these three pathway variables account for 73.20% of the total association in the pathway sample. This pattern suggests that the association between physical activity and subjective class identity is largely reflected through embodied, affective, and relational experiences.

Among the three pathway variables, subjective wellbeing contributes the largest share of the explained component. This result is substantively meaningful because subjective class identity is itself an evaluative judgment. Subjective wellbeing captures people's overall evaluation of life satisfaction and emotional experience (Diener, 1984), and previous studies have linked physical activity to higher wellbeing and positive psychological outcomes (Liu, 2016; Zheng, 2019; Buecker et al., 2021). People do not place themselves on the social ladder only by calculating income or education; they also consider whether their lives feel secure, satisfying, hopeful, and socially recognized. Regular physical activity may be associated with a more positive evaluation of daily life, and this affective evaluation appears to be closely related to subjective class self-placement.

Self-rated health and social interaction also play important roles. Physical activity has been widely associated with health benefits and better functional outcomes (Piercy and Troiano, 2018; Bull et al., 2020), while health status is closely related to class-related perceptions and subjective wellbeing in Chinese society (Fan, 2020; Feng et al., 2024). Better self-rated health may strengthen perceived control, reduce feelings of vulnerability, and support more positive social comparison. Social interaction may provide opportunities for recognition, belonging, information exchange, and informal support. Previous studies also suggest that sport and physical activity can generate social benefits such as connectedness and social support (Yang et al., 2021; Eime et al., 2013). Taken together, the pathway findings suggest that physical activity is connected to subjective class identity not through a single channel, but through the combined operation of the body, emotional evaluation, and social relations.

At the same time, these results should be interpreted as statistical pathway evidence rather than causal mediation. Because the data are cross-sectional, the temporal ordering among physical activity, health, wellbeing, social interaction, and subjective class identity cannot be fully established. The pathway analysis is therefore best understood as identifying theoretically meaningful associations that help clarify why regular physical activity and subjective class identity are empirically connected.

### 5.3 Heterogeneity and the Chinese context

The heterogeneity analysis shows that the positive association between regular physical activity and subjective class identity appears across multiple social groups. Significant positive coefficients are observed among men and women, urban and non-urban respondents, unmarried and married respondents, and several regional and educational groups. This suggests that the association is not limited to one narrow population segment.

However, formal interaction tests indicate that most subgroup differences are modest. Gender, household registration, region, and education do not show statistically significant interaction effects. The clearest difference appears by marital status: the association between regular physical activity and subjective class identity is stronger among unmarried respondents than among married respondents. One possible interpretation is that leisure-time physical activity may play a more visible role in identity formation among unmarried individuals, whose social status evaluation may rely less on household-based roles and more on personal lifestyle, social networks, and individual self-development. Previous studies have shown that marriage and family roles can shape exercise time, physical activity participation, and lifestyle routines (Nomaguchi and Bianchi, 2004; Sobal and Hanson, 2010; Yuan et al., 2024). For married respondents, subjective class identity may be more strongly embedded in household income, family responsibilities, housing, and children's educational prospects, which could reduce the relative salience of individual leisure practices.

The Chinese context is important for interpreting these findings. Physical activity participation is shaped by uneven regional development, the household registration system, income differences, educational resources, and unequal access to public sports facilities. These structural conditions influence who has time, space, and resources to participate in regular leisure-time physical activity (Wang, 2018; Wang and Zhou, 2023; Fang and An, 2024). At the same time, community-based exercise, public parks, square dancing, walking groups, and other low-cost forms of physical activity provide accessible spaces for health maintenance, social interaction, and everyday recognition (Yang et al., 2021). This dual character is important: physical activity reflects social inequality, but it may also create everyday opportunities for social connection and positive self-evaluation.

Therefore, the findings should not be read as suggesting that physical activity can independently overcome class inequality. Instead, they indicate that leisure-time physical activity has social as well as health-related significance. Improving access to safe, inclusive, and affordable physical-activity spaces may matter not only for public health, but also for residents' everyday wellbeing, social participation, and subjective experience of social position.

### 5.4 Limitations

This study has several limitations. First, the data are cross-sectional, so reverse causality and omitted-variable bias cannot be ruled out. People with higher subjective class identity may also be more likely to participate regularly in physical activity because they have more time, resources, confidence, or access to facilities. Second, all key variables are self-reported and may be affected by reporting error or social desirability bias. Third, the five-category subjective class identity measure and the binary physical-activity measure improve interpretability but reduce granularity. Robustness checks using the original 10-point ladder and alternative physical-activity measures reduce this concern but do not eliminate it. Fourth, the pathway analysis and KHB decomposition identify statistical pathway associations rather than causal mediation. Future research should use longitudinal datasets that repeatedly measure leisure-time physical activity, subjective class identity, self-rated and functional health, subjective wellbeing, and social interaction among the same respondents. Panel fixed-effects models could assess whether within-person changes in physical activity are followed by changes in subjective class self-placement, while cross-lagged or related longitudinal designs could compare the temporal pathway from physical activity to subjective class identity with the reverse pathway. These designs would help distinguish temporal sequencing from stable between-person differences. Experiments or defensible external instruments could provide additional causal leverage. Fifth, the CGSS measure of physical activity does not distinguish between type, intensity, duration, and setting of exercise, which are important dimensions for future research.

## 6 Conclusion

Using CGSS 2021 data, this study finds that regular leisure-time physical activity participation is positively associated with subjective class identity among Chinese residents. The main ordered logit model estimates an odds ratio of 1.26 for regular participants, and the association remains robust across alternative variable measurements, model specifications, survey weights, and observed-selection checks. Pathway models and KHB decomposition suggest that self-rated health, subjective wellbeing, and social interaction jointly account for a substantial part of the association, with subjective wellbeing contributing the largest share.

The study extends the literature on social stratification by showing that subjective class identity is connected to everyday practices through embodied, affective, and relational processes. The policy implication is not that physical activity can overcome structural inequality by itself. Rather, equal access to safe, affordable, and socially inclusive physical-activity opportunities may have social as well as health value. Community sports facilities, public exercise spaces, and programs that combine physical activity with social participation and wellbeing promotion may help residents improve daily life while strengthening social connectedness.

## Data Availability

The data analyzed in this study are from the 2021 Chinese General Social Survey (CGSS). Information on the survey and data access is available through the official CGSS website (https://cgss.ruc.edu.cn/). Access to and use of the data are subject to the data provider's requirements and terms of use.
